# Deficient *Lmna* in fibroblasts: an emerging role of non-cardiomyocytes in DCM

**DOI:** 10.20517/jca.2022.26

**Published:** 2022-07-08

**Authors:** Xinjie Wang, Weijia Luo, Jiang Chang

**Affiliations:** Center for Genomic and Precision Medicine, Texas A&M University, Institute of Biosciences and Technology, Houston, TX 77030, USA.

*LMNA* gene encodes intermediate filament proteins Lamin A/C. Lamin A and Lamin C polymerize to form nuclear lamina, mainly located in the inner layer of the nuclear envelope. As an essential component of the nuclear envelope, Lamins are necessary for nuclear structural integrity and participate in chromatin organization, cell cycle regulation, and DNA damage response^[1]^. By far, *LMNA* has the largest and most diverse number of disease-related mutations in the human genome^[2]^. More than 450 different mutations are linked to laminopathies among organs and tissues, including peripheral nerve (Charcot-Marie-Tooth neuropathy), adipose tissue (familial lipodystrophy), muscle tissue (muscular dystrophy, dilated cardiomyopathy, and arrhythmia), multiple systems with accelerating aging (Hutchinson-Gilford progeria syndrome)^[3]^. Most *LMNA* mutations affect the striated muscles; about 165 *LMNA* mutations have been associated with dilated cardiomyopathy (DCM)^[4]^.

DCM is one of the most common causes of heart failure and sudden cardiac death in the aging population, characterized by ventricular dilation and systolic dysfunction. Notably, gene mutation contributes to approximately 40% of DCM causations, and more than 60 genes, including *LMNA*, have been identified as relevant to DCM. *LMNA* mutations lead to 5%−10% of DCM cases, with an age-related penetrance typically between 30 and 40^[5]^. 86% of *LMNA* mutation carriers exhibit cardiac phenotypes over 40, while 7% of the patients are younger than 20^[4]^. *LMNA*-related DCM usually presents conduction defect and ventricular arrhythmia, with or without progression into left ventricular enlargement^[6]^. However, how *LMNA* mutations are linked to the DCM phenotype remains unclear.

Several murine models have been created to unravel the pathogenic signaling pathways that connect the genotype to phenotype [Figure 1]. Growth retardation, cardiac arrhythmias, increased heart fibrosis, early disease onset, and even premature death are shared among Lamin A/C null (*Lmna*^−/−^) mice, mice carrying *Lmna* point mutations (*Lmna*^*N195K/N195K*^ mice, cardiac-specific mutation *Myh6-tTA:tetO-Lmna*^*D300N*^ mice), and cardiac-specific knockout mice (*Myh6-Cre:Lmna*^*F/F*^ mice)^[7-9]^. Interestingly, heterozygous lines with listed mutations grow normally at an early stage but progressively develop DCM at about 12 months. Another systemic mutation-introduced mouse line, *Lmna*^*H222p/H222P*^, exhibited DCM in adulthood, while heterozygous mice lived comparably to *wild-type* mice^[10]^. Transcriptomic analyses have provided clues to illustrate the pathogenic mechanisms. Abnormal activation of MAPK signaling (ERK1/2, JNK, and p38α) and AKT/mTOR signaling has been found in *Lmna*^*H222P/H222P*^ mice, related to the intolerance to energy deficits and decompensation^[10]^. Upregulation of TGF-β signaling in the *Lmna*^*H222P/H222P*^ mouse heart accounts for the myocardial fibrosis. Genes involved in apoptosis, pro-inflammatory cytokines, DNA damage response, and senescence were upregulated upon the *Lmna* variant-driven activation of TP53 in *Myh6-tTA:tetO-Lmna*^*D300N*^ mice^[9]^. In *Lmna*^*N195K/N195K*^ mice, remodeled connexin expression 40/43 on the lateral surface of cardiomyocytes may impair the gap junction communications, while decreased expression of Hf1b/Sp4 in ventricles may affect the cardiac conduction system^[8]^.

In addition to *in vivo* models, patient-specific induced pluripotent stem cell-derived cardiomyocytes (hiPSC-CMs) have been developed as a new and popular model to mimic human diseases and provide the possibility of precision medicine. Patient-derived hiPSC-CMs carrying *LMNA* mutations, especially frameshift mutation, are superior models replicating different DCM-related arrhythmia phenotypes and give the opportunities to elucidate the Lamin regulated-calcium handlings mechanism. A patient-derived hiPSC-CMs carrying *LMNA*^*S143P*^, a prevalent mutation in Finland, have shown increased arrhythmia on β-adrenergic stimulation and are more sensitive to hypoxia^[11]^. Another patient-derived hiPSC-CMs with a frameshift mutation, K117fs, exhibit induced arrhythmias, dysregulation of CAMK2/RYR2 mediated-calcium homeostasis, and electrical abnormalities, consistent with the phenotypes in DCM patients^[12]^. The aberrant activation of platelet-derived growth factor (PDGF) receptor-β (PDGFRB) due to haploinsufficiency of Lamin A/C is considered to contribute to the arrhythmic phenotype in mutant hiPSC-CMs^[12]^.

To date, almost all mechanistic studies on *LMNA*-related DCM have focused on cardiomyocytes, as cardiomyopathy is considered the disease of cardiomyocytes. However, as mentioned at the beginning, *LMNA* is widely expressed. The gene carrying the casual mutations in CMs is also expressed in other cell lineages, such as fibroblasts. Emerging evidence indicated that cardiac fibroblasts and fibrosis play a significant role in DCM pathogenesis. However, a knowledge gap remains on whether cardiac fibroblasts also participate in the occurrence of *LMNA*-associated DCM.

Here, Dr. Marian and his team generated a fibroblast-specific *Lmna* knockout mouse line and demonstrated that the deletion of *Lmna* in cardiac fibroblasts contributes to senescence-related DCM phenotype^[13]^. *Lmna* in fibroblasts was deleted by crossing PDGF receptor-α recombinase (*Pdgfra-Cre*) and floxed *Lmna* (*Lmna*^*F/F*^) mice. Nearly 80% of *Lmna* was absent in cardiac fibroblasts, confirming the knockout efficiency. The *Pdgfra-Cre:Lmna*^*F/F*^ mice showed growth retardation, cardiac conduction defects, arrhythmias, cardiac dysfunction, myocardial fibrosis, increased apoptosis, and premature death within six weeks, recapitulating typical *Lmna*-DCM phenotypes. Significantly smaller body weights and higher mortality observed in *Pdgfra-Cre:Lmna*^*F/F*^ mice are similar to the phenotypes in mice with global or cardiac homozygous Lmna deletion or mutations. Increased LVEDDI and LVMI and decreased LVFS in *Pdgfra-Cre:Lmna*^*F/F*^ mice indicated left ventricular dilation and systolic dysfunction. Parallelly, cardiomyocyte hypertrophy was found in *Lmna* fibroblast-knockout mice. 8/12 of the *Pdgfra-Cre:Lmna*^*F/F*^ mice exhibited various arrhythmias. Interestingly, the heterozygous (*Pdgfra-Cre:Lmna*^*W/F*^) developed a similar but slowly progressing phenotype with disease onset at 12–18 months of age, correlating to an age-associated penetrance in patients and dosage-dependency of laminopathy. Moreover, increased collagen volume fraction combined with elevated TGFβ1 implied progressive myocardial fibrosis in *Lmna*-deficient mouse hearts. Given that fibroblasts can crosstalk with cardiomyocytes via paracrine signals, extracellular matrix remodeling, and electric coupling, *Lmna*-deficient fibroblasts may account for those cardiac phenotypes^[14]^. Escalation of apoptosis was induced by *Lmna* knockout in conjunction with activation of DNA damage response, likely due to the damaged structure of the nucleus envelope due to Lamin A/C deficiency. Noteworthily, a senescence-associated secretory phenotype (SASP) was discovered in fibroblast-specific *Lmna*-deleted mice. Incorporating with studies on cardiomyocytes, *Lmna* deletion in fibroblasts displayed a cohesive phenotype and similar pathogeneses, implying a potential synergetic mechanism between cardiac myocytes and fibroblasts.

In this paper, the authors elaborately defined the progressive DCM phenotype, thoroughly demonstrating the potential role of fibroblasts in *LMNA*-DCM, and proposed underlining mechanisms. These findings are remarkable as presenting the first direct evidence that fibroblasts participate in the *LMNA*-regulated DCM pathogenesis. The study provides a new angle on delineating the underlying mechanism for DCM with *LMNA* mutations. That is, non-cardiomyocytes such as cardiac fibroblasts, and cardiomyocytes, jointly contribute to DCM pathogenesis. This research and future investigations on the function of *LMNA* in endothelial and smooth muscle cells will further critical new information for clinical treatments.

A few limitations of the study are noted. As the authors mentioned in the discussion, *Pdgfra* is not a unique marker for cardiac fibroblasts. The *Pdgfra* promoter-driven Cre expression may lead to partial deletion of *LMNA* in other cell types and tissues, eventually affecting the net phenotype. To exclude the effect of non-cardiac-fibroblast *LMNA* deletion, the authors screened the comprehensive metabolic panel, which suggested no apparent abnormalities in liver or kidney, and electrolyte disturbances, except a reduced glucose level in plasma. Interestingly, around 25% of cardiomyocytes showed *LMNA* deletion in *Pdgfra-Cre:Lmna*^*F/F*^ mice. However, *Pdgfra-Cre:Lmna*^*W/F*^ mice with intact Lamin A/C expression in cardiomyocytes still developed DCM with a late-onset, implying a fibroblast-independent role in the disease progression. While the lack of highly specific markers for cardiac fibroblasts remains a challenge in the field, the inducible *Tcf21-MerCreMer* could serve as an alternative approach to confirm the fibroblast contribution to DCM^[15]^. Additionally, *Lmna* can be ablated in active myofibroblasts by *Postn-MerCreMer* to further elucidate the role of fibroblast *LMNA* in other comorbid cardiovascular diseases such as myocardial infarction and diabetic cardiomyopathy^[16,17]^.

In summary, the current study filled the knowledge deficiency of cardiac fibroblasts in developing *LMNA* deficiency-induced cardiomyopathy. The new information expands the understanding of the pathogenesis of the *LMNA*-induced DCM.

## Figures and Tables

**Figure 1.**
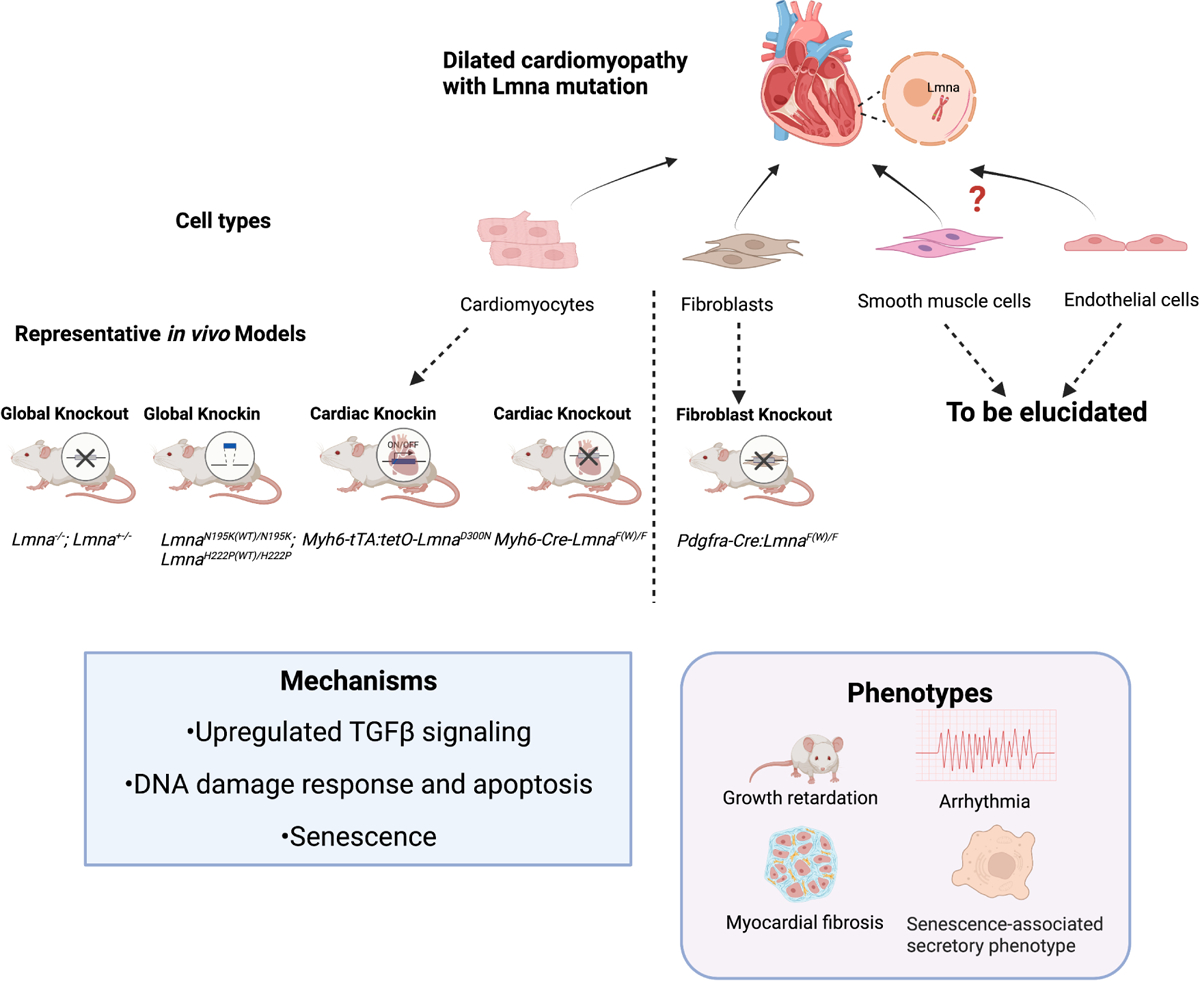
Patient-related *LMNA* mutant mouse models. Most animal models are generated based on *Lmna* mutation or deletion in cardiomyocytes and have contributed to investigations on DCM pathogenesis. The current fibroblast-specific *Lmna*-deficient mice demonstrated a similar DCM phenotype compared to cardiomyocyte *Lmna*-deficient models, with the signature of growth retardation, arrhythmia, and myocardial fibrosis, and senescence-associated secretory phenotype. Mechanistic studies showed upregulation of TGF β signaling, activation of DNA damage response and apoptosis, and cell senescence. Collectively, cardiac fibroblasts with *Lmna* deficiency jointly contribute to DCM with cardiomyocytes. With this discovery, the non-cardiomyocytes are emerging as important new players in the pathogenesis of *LMNA*-DCM. (Created with BioRender.com).
